# Supplementary material for: Comparative Clustering (CompaCt) of eukaryote complexomes identifies novel interactions and sheds light on protein complex evolution
Source: PLoS Comput Biol. 2023 Aug 7;19(8):e1011090. doi: 10.1371/journal.pcbi.1011090 (PMC10434966; doi:10.1371/journal.pcbi.1011090)
Supplement: S1 Appendix — (PDF) [file pcbi.1011090.s001.pdf]

S1 Appendix

Comparative Clustering (CompaCt) of Eukaryote complexomes identifies novel interactions and sheds light on protein complex evolution

Joeri van Strien[1], Felix Evers[2], Madhurya Lutikurti[3], Stijn L. Berendsen[3], Alejandro Garanto[3,4,5], Geert-Jan van Gemert[2], Alfredo Cabrera-Orefice[1], Richard J. Rodenburg[5,6], Ulrich Brandt[3,5,7], Taco W.A. Kooij[2], Martijn A. Huynen[1*]

[1] Medical BioSciences Department, Radboud University Medical Center, Nijmegen, the Netherlands

[2] Medical Microbiology, Radboud Center for Infectious Diseases, Radboud University Medical Center, Nijmegen, The Netherlands.

[3] Department of Pediatrics, Amalia Children's Hospital, Radboud University Medical Center, Nijmegen, the Netherlands

[4] Department of Human Genetics, Radboud University Medical Center, Nijmegen, The Netherlands

[5] Radboud Center for Mitochondrial Medicine (RCMM), Radboud University Medical Center, Nijmegen, the Netherlands

[6] Department of Pediatrics, Translational Metabolic Laboratory, Radboud University Medical Center, Nijmegen, the Netherlands

[7] Cologne Excellence Cluster on Cellular Stress Responses in Aging-Associated Diseases (CECAD), University of Cologne,Cologne, Germany

* Martijn.Huijnen@radboudumc.nl

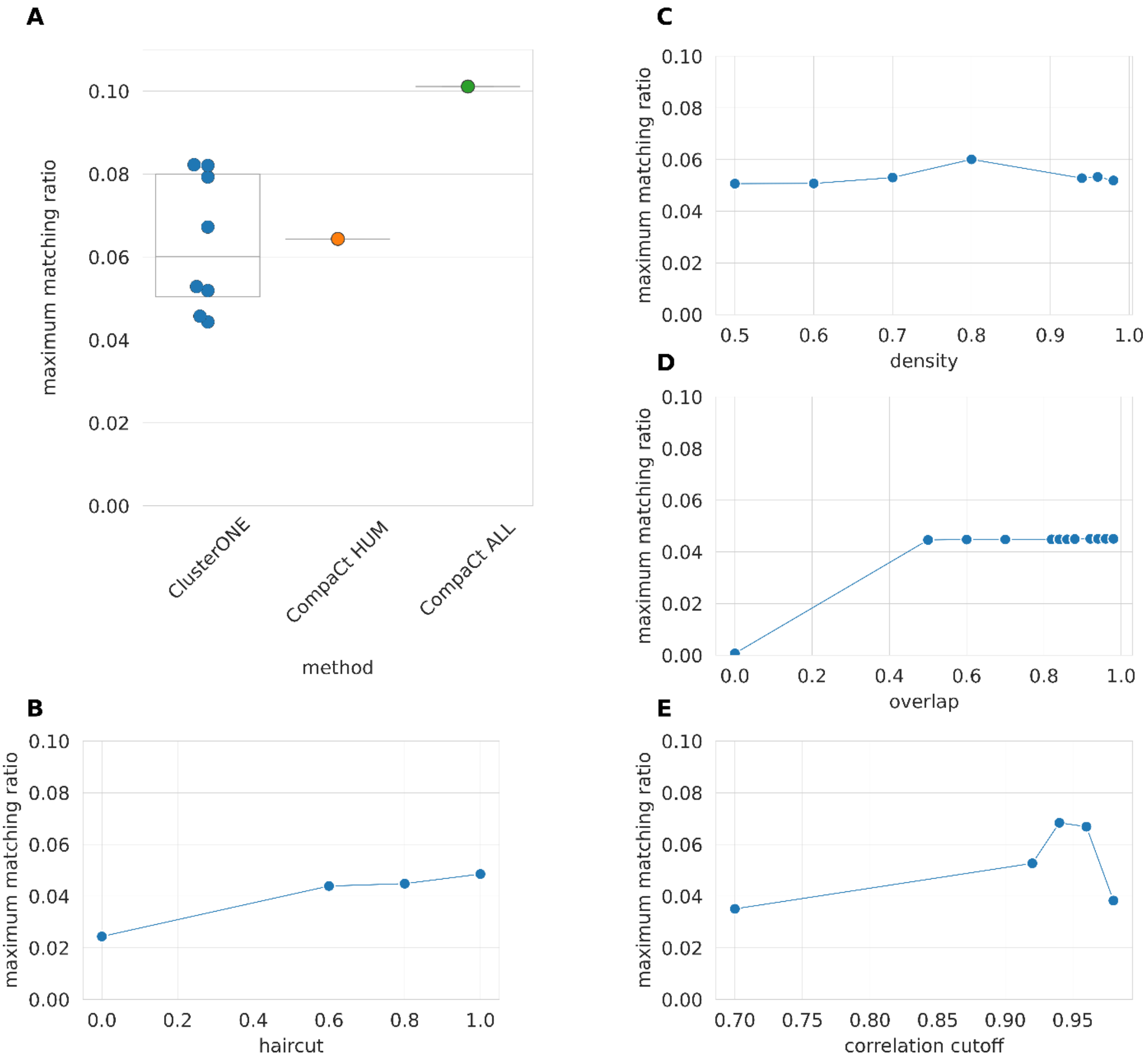

Fig A. A) agreement of cluster results with CORUM [1] reference complexes, after clustering human complexome profiles with ClusterONE [2] and CompaCt. Pairwise pearson correlations were computed from the complexome profiling data, and used as input for both ClusterONE and CompaCt. ClusterONE was applied and its performance evaluated for each separate complexome profile. Agreement of cluster results is quantified using the maximum matching ratio. CompaCt results are shown when applied to the eight human complexome profiles (“CompaCt HUM”), as well as to the complete dataset including all complexomes (“CompaCt ALL”). B-D) Performance for different ClusterONE parameters when applied to CRS17, when keeping the other parameters fixed. The highest performing parameter values were used for the performance shown in panel A. E) the performance of the tool using various correlation score thresholds for inclusion of the edge in the ClusterONE input network. The x-axis shows the minimum correlation score that is included.

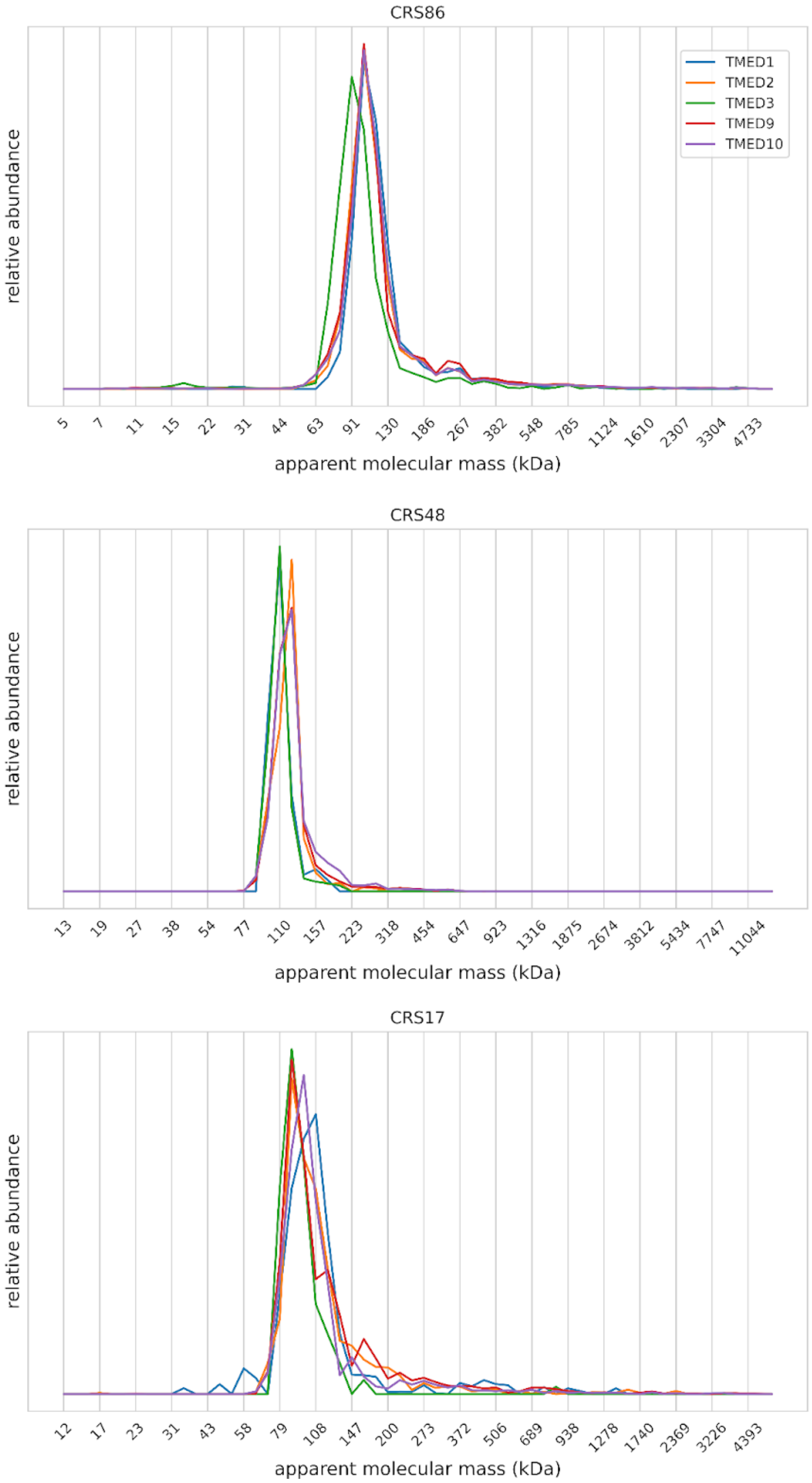

Fig B. Migration of clustered *H.sapiens* EMP24 proteins in three human fibroblast complexome profiles. The apparent mass calibration of the complexome profiling samples was taken from the publications that first presented these data [3–5]. Relative abundances were obtained by scaling the iBAQ values to a unit vector for each protein. The major peak of these TMED protein's migration patterns overlap at an apparent molecular mass of 100 to 120 kDa, corresponding approximately to the combined mass of these proteins: 125 kDa.

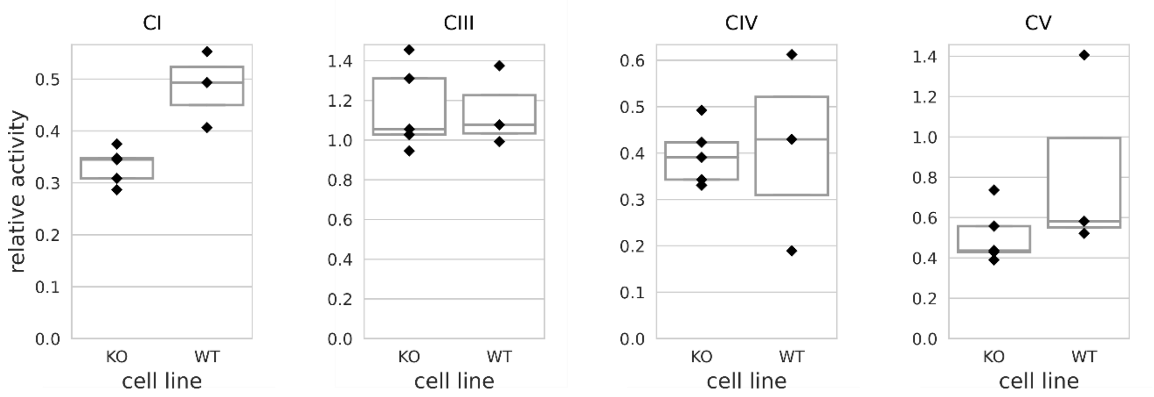

Fig C. Enzyme activities in C15orf61-KO and WT HEK cell lines, as measured by spectrophotometric analysis. The enzyme activity values are relative to the activity of respiratory chain complex II. No significant changes were observed but complex I activities tended to be lower in the KO cell lines (Bonferroni adjusted p-values; CI: 0.092, CV: 0.583, Mann-Whitney U test). The boxes indicate quartiles and the horizontal line the median value.

```
                              cov    pid   1 [         .         .         .     ] 36
 1 Homo                    100.0% 100.0%     SFFSPGIWMGLLTSLFMLFIFTYGLHMILSLKTMDR
 2 Anopheles               100.0%  38.9%     GFTSGGILSGLFLILIFIIIGSYGIAWMMDIRTMDR
 3 Ectocarpus              100.0%  33.3%     IKITPDILAGLLTLLLFLFILVTGLGCVGDIECPKS
 4 Saccharomyces           100.0%  30.6%     SIWTEGLLMCLIVSALLLFILIVALSWISNLDITYG
 5 Naegleria               100.0%  27.8%     LMIDGEIAAGTLVGLFLLFFLVIAIVFLMNLKTSPH
 6 Arabidopsis             100.0%  25.0%     CKFKSSLLEGILVGIVFLLILISGLCCMAGIDTPTR
 7 Capsaspora              100.0%  22.2%     TWWTIPILMGLFVGAILFSILLTGVISIFTTETPTK
 8 Schizosaccharomyces     100.0%  22.2%     QFFTPGLYMGYLALAVLVPTLFISCRLLSSIQISYH
 9 Dictyostelium           100.0%  22.2%     TYVTGPVLSAYLIISILLAILFTGICCISDLQVPDR
10 Physcomitrella          100.0%  16.7%     CRSRAVILEGIFVAFTLITILVSGICCMKAVKSPAR
11 Plasmodium              100.0%  16.7%     FHTNPNILSQLMIVFLIFFFLFIGFYVLINISTPKI
12 Tetrahymena             100.0%   5.6%     QIPNQTLFGLILVFILFIPVLWIGISCLYGIESPEK
13 Toxoplasma              100.0%   2.8%     KYVTSTMLSQVVVIFLIAVTAAVGVSCLSNIDVPEI
   consensus/100%                            .h.p..hh..hhh..hhh.hh..uh..h.shp....
   consensus/90%                             .h.p.slh.thhh.hhhh.hhh.uh.hh.slp...h
   consensus/80%                             .hhs.slh.thhhhhhhh.hhhhul.hh.slpss.t
   consensus/70%                             thhssslh.slhlhhlhlhhLhhGlthl.slcsstp
```

Fig D. Alignment of the conserved C-terminus of human ATP6AP1 and its predicted orthologs in a number of species. The *A. stephensi* (A0A182XX75) and *P. falciparum* (PF3D7_0713700) orthologs aligned here are part of the supercluster representing the V_0 component of the vacuolar ATPase complex resulting from our analysis. Multiple sequence alignment was performed with ClustalOmega v1.2.4 [6,7] using default settings, and the alignment was visualized with Mview v1.63 [8].

```
Reference sequence (1): sp|P24311|COX7B_HUMAN
Identities normalised by aligned length.
Colored by: identity


                                    cov     pid   1 [         .         .         .         .         :         .         .         . 80
1 sp|P24311|COX7B_HUMAN          100.0% 100.0%      -------MFPLVKSALNRLQVRSIQQTMARQSHQKR---------------------TPDF------HDKYGNAVLASGA
2 tr|Q9VVG5|Q9VVG5_DROME          80.0%  11.1%      ----------------MLVKHIVKQGLLLKNAGALSRAAYHGG-HGPHSTMNDLPVPAGDWKEQHSQKNAKYNAALITGI
3 tr|A0A182Y1F7|A0A182Y1F7_ANOST  91.2%  16.0%      LQTVQYLRFSALQETKKMQPVRSVANLAIRNAAFLSR-GYHGPSNFRVYTMNDMPVPEGDFFEEHRRKNRVYNTVLAAGI
  consensus/100%                                    .................h..hhshtthhh+put.hp.....................tsDa......+sthhNssLhsGh
  consensus/90%                                     .................h..hhshtthhh+put.hp.....................tsDa......+sthhNssLhsGh
  consensus/80%                                     .................h..hhshtthhh+put.hp.....................tsDa......+sthhNssLhsGh
  consensus/70%                                     .................h..hhshtthhh+put.hp.....................tsDa......+sthhNssLhsGh

                                    cov     pid  81         .         1         .   ] 114
1 sp|P24311|COX7B_HUMAN          100.0% 100.0%      TFCIVTWTYVATQVGIEWNLSPVGRVTPKEWRNQ
2 tr|Q9VVG5|Q9VVG5_DROME          80.0%  11.1%      LVLAGTIGFVKSSGIIHFNYYAPKSLD-------
3 tr|A0A182Y1F7|A0A182Y1F7_ANOST  91.2%  16.0%      VIFGITLTVAKESGLIYLNYNPPKSLD-------
  consensus/100%                                    hhhhhThshstppshI.hNh.sstpls.......
  consensus/90%                                     hhhhhThshstppshI.hNh.sstpls.......
  consensus/80%                                     hhhhhThshstppshI.hNh.sstpls.......
  consensus/70%                                     hhhhhThshstppshI.hNh.sstpls.......
```

Fig E. Alignment of COX7B with recently identified *D. melanogaster* ortholog C9VVG5 and *A. stephensi* A0A182Y1F7 protein that clusters with Complex IV in our results. Multiple sequence alignment was performed with ClustalOmega v1.2.[6,7] using default settings, and the alignment was visualized with Mview v1.63 [8].

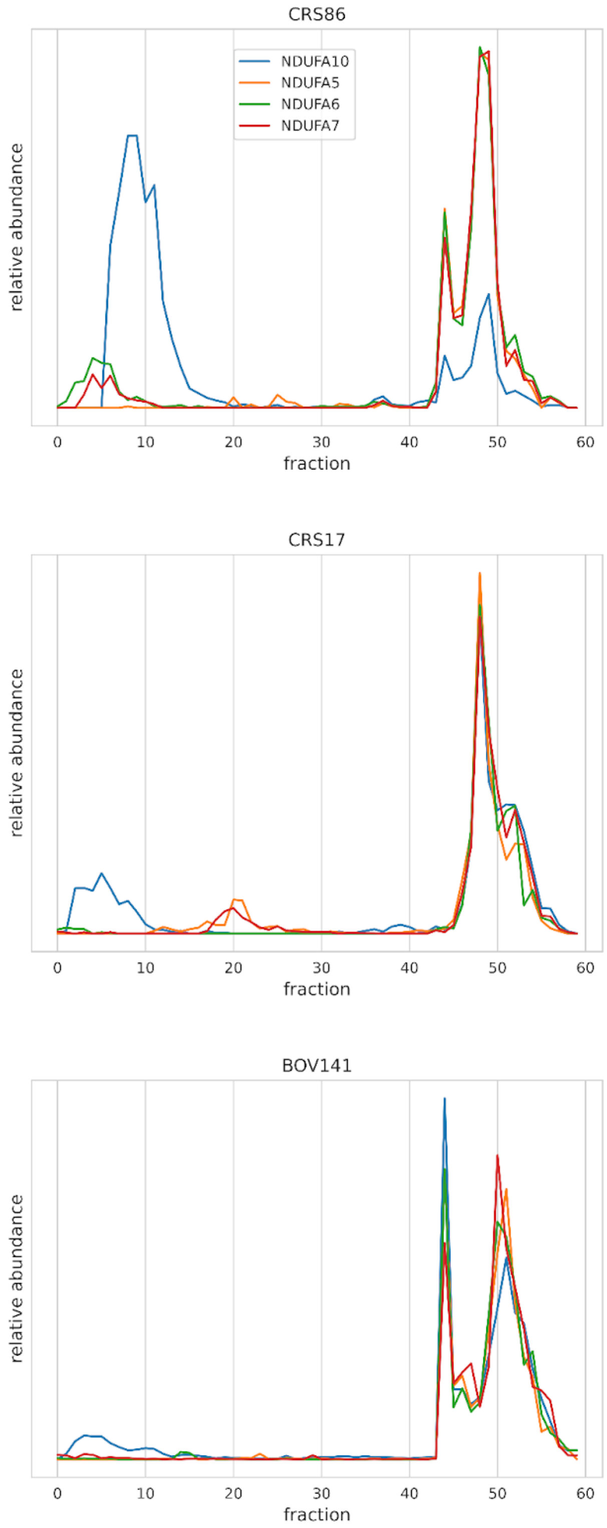

Fig F: Migration of four complex I proteins in two *H. sapiens,* one *B. taurus* and one *A. stephensi* complexome profiles. Relative abundances were obtained by scaling the iBAQ values to a unit vector for each protein. Aside from its migration together with the other complex I subunits, it also occurs at lower abundance, suggesting that aside from being part of complex I, it is present in the samples as a monomer.

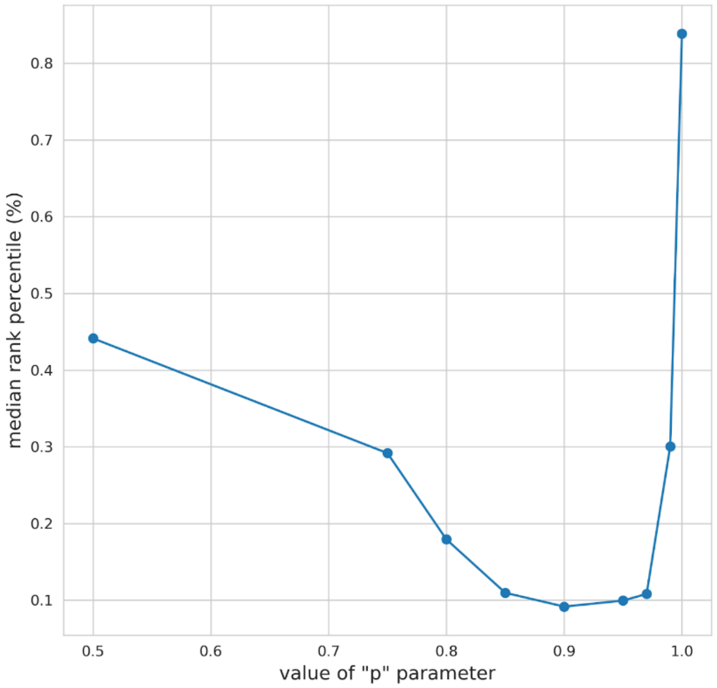

Fig G. performance for a range of rank biased overlap (RBO) “p” parameter values for identification of proteins that are part of the same protein complex. The p parameter determines the “top heaviness” of the RBO metric, i.e., the degree to which higher ranks influence the RBO score more than lower ranks when comparing ranked lists of protein interactions. To determine the optimal RBO p parameter for the identification of pairs of proteins that are part of the same complex, RBO scores were computed for all possible protein pairs between two complexome profiles, one from *P. falciparum* and one from *T. gondii*. An evaluation set of 1285 protein pairs was created, each consisting of one *Toxoplasma* and one *Plasmodium* protein that are known to be part of the same protein complex, using a set of protein complexes conserved between these species. After ranking all protein pairs based on their RBO scores, the median rank of the evaluation set was computed, expressed in a percentile of the total list, to represent the degree to which the RBO metric scores protein pairs that are part of the same protein complex higher than other protein pairs. The median rank of the evaluation set was computed for a range of different values for “p”.

Table A. Overview of 15 inspected *A. stephensi* subclusters

Supercluster id	Complex	Predicted association	Not associated, subunit ortholog	Unknown function
1	Complex I	30	2	1
10	Complex II	2	0	1
8	Complex III	3	1	1
9	Complex IV	4	0	1
3	Complex V	14	0	3
4	20S Proteasome	6	0	0
7	V-type ATPase V0	7	0	3
13	V-type ATPase V1	6	0	0
15	Prohibitin	2	0	1
16	Oxoisovalerate Dehydrogenase	1	1	1
26	Dolichyl-diphosphooligosaccharide—protein glycosyltransferase	3	0	0
28	Signal peptidase	3	0	1
30	60S acidic ribosomal proteins	2	1	0
31	Electron transfer complex	2	0	0
97	Integrin complex	3	0	0
**Total**	15	88	5	13

Overview of 15 inspected *A. stephensi* subclusters resulting from CompaCt analysis of eukaryote complexome profiling data, filtered using the "best guess" cluster member selection criterion. Protein members are divided in three categories based on information available in their Uniprot [9] entries: proteins that have annotations associating them with the respective protein complex, proteins whose function is unknown, and proteins that have no evidence associating them with the complex, but we predict based on sequence-based homology to be orthologous with a known subunit of the respective complex.

Table B. Composition of supercluster 410, containing the rubisco complex

	AT_LF			AT_SD		
	**CRS100**	**CRS101**	**CRS102**	**CRS103**	**CRS104**	**CRS105**
**size**	4	7	3	8	12	6
**members**	AT5G50920<br>AT1G67090<br>ATCG00490<br>AT3G01910	AT4G39080<br>ATCG00490<br>AT1G67090<br>AT5G38420<br>AT5G12290<br>AT1G52230<br>AT2G20990	ATCG00490<br>AT5G38420<br>AT1G67090	AT4G30040<br>AT1G71210<br>AT2G34520<br>ATCG00490<br>AT5G38420<br>AT1G67090<br>AT5G38430<br>AT4G32130	AT5G02490<br>AT5G38430<br>AT5G38420<br>AT1G67090<br>ATCG00490<br>AT4G30040<br>AT5G22510<br>AT3G28720<br>AT4G30340<br>AT5G12290<br>AT5G50990<br>AT2G20990	AT5G38420<br>AT1G67090<br>AT5G38430<br>AT2G27690<br>ATCG00490<br>AT3G44330

Table C. Overview of complexome profiles used in main analysis

Complexome Alias	species	description	CEDAR experiment ID	samples used	CEDAR sample id(s)
HUM	*H. sapiens*	fibroblast mitochondria	CRX22	1	CRS86
HUM	*H. sapiens*	fibroblast mitochondria	CRX17	1	CRS50
HUM	*H. sapiens*	fibroblast mitochondria	CRX15	1	CRS48
HUM	*H. sapiens*	fibroblast mitochondria	CRX9	4	CRS22-25
HUM	*H. sapiens*	fibroblast mitochondria	CRX8	1	CRS17
BOVIN	*B. taurus*	heart mitochondria	CRX33	6	CRS139-144
ANOST	*A. stephensi*	salivary tissue	CRX41	2	CRS201-203
YARLI	*Y. lipolytica*	mitochondria	CRX40	4	CRS197-200
PF3_GAM	*P. falciparum*	gametocyte mitochondria	CRX23	4	CRS96-99
PF3_AS	*P. falciparum*	asexual stage mitochondria	CRX23	4	CRS92-95
PF3_SCH	*P. falciparum*	blood-stage schizont whole cell	CRX20	6	CRS70-75
BER_SCH	*P. berghei*	blood-stage schizont whole cell	CRX20	6	CRS58-63
KNO_SCH	*P. knowlesi*	blood-stage schizont whole cell	CRX20	6	CRS64-69
TOX	*T. gondii*	tachyzoite mitochondria	CRX27	1	CRS114
AT_LF	*A. thaliana*	leaf mitochondria	CRX24	3	CRS100-102
AT_SD	*A. thaliana*	seedling mitochondria	CRX24	3	CRS103-105

Table D. Overview of proteomes used for sequence-based homology analysis

species	proteome description	source	Reference
A. thaliana	Araport11_pep_20210622_representative_gene_model	https://www.arabidopsis.org	[10]
H. sapiens	UP000005640_9606	uniprot.org	[9]
B. taurus	UP000009136_9913	uniprot.org	[9]
A. stephensi	UP000076408_30069	uniprot.org	[9]
Y. lipolytica	UP000001300_284591	uniprot.org	[9]
P. falciparum	PlasmoDB-52_Pfalciparum3D7_AnnotatedProteins	https://plasmodb.org/	[11]
P. knowlesi	PlasmoDB-55_PknowlesiH_AnnotatedProteins	https://plasmodb.org/	[11]
P. berghei	PlasmoDB-55_PbergheiANKA_AnnotatedProteins	https://plasmodb.org/	[11]
T. gondii	ToxoDB-52_TgondiiME49_AnnotatedProteins	https://toxodb.org/	[11]

SUPPLEMENTAL METHODS

ClusterONE analysis of human complexome profiles

To determine the performance of ClusterONE (v1.0) [2], it was applied to the eight *H. sapiens* complexome profiling datasets, using the parameter settings that yielded the highest performance. The optimal ClusterONE parameters were determined by computing the agreement of cluster performance for various parameters for a range of values for each parameter, while keeping the other parameters fixed (Fig A). The optimal parameter settings were as follows: overlap=0.96, density=0.8, haircut=1.0. As the penalty parameter did not have a significant effect on the performance it was kept at its default value of 2. In addition to the aforementioned ClusterONE algorithm parameters, different threshold values for inclusion of correlation score edges in the ClusterOne input were evaluated (Fig A). Retaining only edges with a correlation score of at least 0.96 resulted in the best performance.

Generation of *P. falciparum* sporozoites / *A. stephensi* salivary gland material

P. falciparum strain NF54 asexual blood stage parasites were maintained in RPMI supplemented with 10% human serum and 5% haematocrit using standard culturing technique [12]. To induce gametocytogenesis, asexual parasites were set at 0.5% parasitemia and left to overgrow for 14 days in a semi-automated shaker system until harvest [13]. 900 µL of the gametocyte culture (approximately 2.5% haematocrit and 1-8% gametocytemia) was mixed with 540 µL packed RBCs and the cells were centrifuged for 20s at 600 x G. The supernatant was carefully removed and the pellet resuspended in 450 µL of human serum. Midi membrane feeders were used to feed female *A. stephensi* (Sind Kasur Nijmegen strain) mosquitoes [14]. On day 17 after feeding, mosquitoes were dissected and the salivary glands collected and homogenized with home-made glass grinders in complete Williams B media [William's E medium with Glutamax (Thermo Fisher, 32551-087),

supplemented with 1× insulin/transferrin/selenium (Thermo Fisher, 41400-045), 1 mM sodium pyruvate (Thermo Fisher, 11360-070), 1× MEM-NEAA (Thermo Fisher, 11140-035) at room temperature [15]. Sporozoites were counted in a Burker-Turk chamber using phase contrast microscopy.

Separation of *P. falciparum* sporozoites / *A. stephensi* salivary gland material

The following steps were performed at RT with all solutions brought to RT prior to usage unless mentioned otherwise. 20% w/v Accudenz (Accurate Chemical #AN7050) was made up in demiwater and supplemented with 1× cOmplete™ EDTA-free Protease Inhibitor Cocktail (Sigma) and filter sterilized. 4.5mL were added to a conical 15mL tube. Sporozoite / salivary gland homogenate was suspended in a total of 2mL Williams B medium and carefully layered on top of the Accudenz cushion. The tube was centrifuged for 20 minutes at 2500 x G without brake. Sporozoites were extracted from top of Accudenz cushion, while salivary gland material was recovered from bottom of the tube. Both fractions were washed twice in 4°C PBS supplemented with 1× cOmplete™ EDTA-free Protease Inhibitor Cocktail and the dry pellets were flash frozen and stored at -80°C. For the non-separated sample all steps prior to washing were skipped.

Blue native polyacrylamide gel electrophoresis (BN-PAGE)

Protein samples were resuspended in 500 mM 6-aminohexanoic acid, 1 mM EDTA and 50 mM imidazole/HCl (pH 7.0). The samples that were separated into sporozoite and gland material were solubilized with n-dodecyl-β-D-maltoside (DDM) (Sigma) using a detergent:protein (w/w) ratio of 3:1, while the non-separated sporozoite / salivary gland homogenate was solubilized with digitonin (SERVA) at a detergent:protein (w/w) ratio of 6:1. The solubilized samples were centrifuged at 22,000 × *g* for 20 min; 4 °C. The supernatants were recovered, supplemented with Coomassie-blue loading buffer and separated on either a 4–16% or 3–16% polyacrylamide gradient blue native gels as described previously[16].

Cell culture conditions

HEK293T cells (ATTCC, 293T-CRL-3216) were cultured in High glucose DMEM medium supplemented with 10% fetal calf serum, 1% penicillin/streptomycin and 1% sodium pyruvate. Cells were cultured at 37 °C and 5% CO_2.

Generation of KO HEK293T cells

Two different gRNAs were designed using CRISPOR (http://crispor.tefor.net, last accessed 22 November 2022) and CHOPCHOP (https://chopchop.cbu.uib.no, last accessed 22 November 2022). Sequences of the gRNA are provided in Table S4. Two different strategies were followed: First one consisted of designing a gRNA close to the codon of the initial methionine of *C15orf61* (gRNA1). The other strategy aimed to excise a big part of the gene using two gRNAs (gRNA1 and gRNA2). gRNAs were cloned into the pSpCas9(BB)-2A-GFP (PX458) vector (Addgene, Plasmid #48138) following the protocol previously described [17]. Subsequently, plasmids were transfected into HEK293T to evaluate efficacy using FuGene (Promega) reagent in a 1:3 ratio following manufacturer's instructions. After 24 h, cells fluorescent indicating that the transfection worked. Cells were harvested after 48 h and subjected to DNA isolation by incubating the pellets with MQ for 10 min at 95 °C, then adding proteinase K for 20 min at 56 °C, followed by 5 min at 95 °C to inactivate it. A standard 15 µl AmpliTaq (Life Technologies, 4398881) PCR using the primers for each of the regions (Table S5) was set as follows: 10 min at 95 °C, followed by 35 cycles of 60 sec at 95 °C, 60 sec at 54 °C and 45 sec at 75 °C, with a final elongation step of 5 min at 75°C. Subsequently, samples were loaded onto a 2%-agarose gel and were sent for Sanger sequencing to validate efficacy of gRNAs.

After validating gRNA efficacy, HEK293T at passage 20 were transfected either with gRNA1 or gRNA1+2. After 48 h, single cell sorting for GFP positive cells was performed, resulting in seeding one cell in each well of a 96 well plate. In total 2 full plates were used per condition. Clones that grew were passed to a 12 well plate, and then to a 6 well plate and a T25. Clones

were sequenced for mutation identification at passage 23. Selected clones with potential promising mutations were expanded. To confirm that the editing was stable cells were sequenced at passage 25, 28 and 30. For the genotyping, for the strategy of only one gRNA, samples were amplified and sequenced using gRNA1 region primers. For the combination of two gRNAs, gRNA1 region Fw and gRNA2 region Rv were used. PCR conditions were the same as the ones described above.

In silico off-target analysis was performed using CRISPOR and CHOCHOP. For gRNA1 and gRNA2 no off-target sequences with an NGG PAM and no mismatches in the adjacent 12 nucleotides were found (Table S4). For gRNA1 an off-target sequence with 3 mismatches in one exon of the *UBALD1* gene was predicted by the two software's (Table S7). For gRNA2, two intergenic regions in chrosome 3 and 5 were predicted as off-target. Given the location of the possible off-targets, we validated only the *UBALD1* region as it was predicted in a gene. The region was amplified using the corresponding primers (Table S5) and by Sanger sequencing. No genome editing was observed in that region for any of the clones for which gRNA1 was used (Table S7).

For the enzyme activity data, the clones in Table S6 were used. Briefly, cells from a T75 were pelleted and frozen at passage 29.

Table E. gRNA sequences and off-target determined by CRISPOR and CHOCHOP

gRNA	Sequence 5' to 3'	PAM site	CRISPOR off-targets with 0-1-2-3 mismatches*	CHOCHOP off-targets with 0-1-2-3 mismatches*
gRNA1	CAGGCGGAGCGCGACCTCGT	GGG	0 - 0 - 0 - 1	0 - 0 - 0 - 1
gRNA2	TGTAGGTTAACCTAGTTCTA	GGG	0 - 0 - 0 - 2	0 - 0 - 0 - 2

Using the most stringent filter (no mismatches in the 12 nucleotides adjacent to the PAM), both gRNA had no off-targets.

Table F Primer sequences used to amplify and sequence *c15orf61* HEK293T KO

Primer	Sequence 5'to 3
gRNA1 region Fw	CAAAACCAGCTCCTTGACGC
gRNA1 region Rv	CAGAAGGAGGTCCAGTGCG
gRNA2 region Fw	ACTCTGTTTAGCTGATGTGAACT
gRNA2 region Rv	TCCTGGCAGCTGAATGGTTT
Off target UBALD1 Fw	AGACCAACATCCCCTACAGC

Off target UBALD1 Rv	GGCTGTAGGGTAAGGAGCTT

Table G. Characteristics of the different *c15orf61* HEK293T clones used in this work

Clone	Alias	Passage	Allele 1	Allele 2	gRNA used
HEK293T not transfected	FM-WT HEK	29	WT	WT	none
Clone 2E7	FM-2-E7	29	WT	WT	gRNA1
Clone 4E7	FM-4-E7	29	WT	WT	gRNA1+gRNA2
Clone 2D4	FM-2-D4	29	c.-8_132delins83 p.Met1?	c.-101_95delins45 p.Met1?	gRNA1
Clone 2E11	FM-2-E11	29	c.-149_103delins149 p.Met1?	c.-149_103delins149 p.Met1?	gRNA1
Clone 3C10	FM-3-C10	29	c.26_*243del p.(Ala11Cysfs*21)	c.26_*243del p.(Ala11Cysfs*21)	gRNA1+gRNA2
Clone 4F5	FM-4-F5	29	c.-152_*267del p.Met1?	c.-152_*267del p.Met1?	gRNA1+gRNA2

Table H. Off-target sequences predicted by CRISPOR and CHOCHOP

gRNA	Off-target sequence	region	Location	Strand	MM	Result
#1	CAGGCGGGGCTCGACCTCTT *TGG*	exon: *UBALD1*	chr16: 4660370-4660392	-	3	No editing found
#2	TGCAGGTTAAACTAGTTCGA *AGG*	intergenic: RP11-154H23.3-EIF4E3	chr3: 71691699-71691721	+	3	Not screened
#2	TGTAGGTTAACCTCCTTCCA *TGG*	intergenic: C5orf66-C5orf66/H2AFY	chr5: 134642107-134642129	-	3	Not screened

Nucleotides in red indicate the mismatches with the original sequence. The PAM sequence is depicted in italics. MM: Mismatches

REFERENCES

1. Giurgiu M, Reinhard J, Brauner B, Dunger-Kaltenbach I, Fobo G, Frishman G, et al. CORUM: the comprehensive resource of mammalian protein complexes—2019. Nucleic Acids Res. 2019 Jan 8;47(D1):D559–63.

2. Nepusz T, Yu H, Paccanaro A. Detecting overlapping protein complexes in protein-protein interaction networks. Nat Methods. 2012 May;9(5):471–2.

3. Alston CL, Veling MT, Heidler J, Taylor LS, Alaimo JT, Sung AY, et al. Pathogenic Bi-allelic Mutations in NDUFAF8 Cause Leigh Syndrome with an Isolated Complex I Deficiency. Am J Hum Genet. 2020 Jan 2;106(1):92–101.

4. Chatzispyrou IA, Guerrero-Castillo S, Held NM, Ruiter JPN, Denis SW, IJlst L, et al. Barth syndrome cells display widespread remodeling of mitochondrial complexes without affecting metabolic flux distribution. Biochim Biophys Acta Mol Basis Dis. 2018 Nov;1864(11):3650–8.

5. Gardeitchik T, Mohamed M, Ruzzenente B, Karall D, Guerrero-Castillo S, Dalloyaux D, et al. Bi-allelic Mutations in the Mitochondrial Ribosomal Protein MRPS2 Cause Sensorineural Hearing Loss, Hypoglycemia, and Multiple OXPHOS Complex Deficiencies. Am J Hum Genet. 2018 Apr 5;102(4):685–95.

6. Madeira F, Pearce M, Tivey ARN, Basutkar P, Lee J, Edbali O, et al. Search and sequence analysis tools services from EMBL-EBI in 2022. Nucleic Acids Res. 2022 Apr 12;50(W1):W276-279.

7. Sievers F, Wilm A, Dineen D, Gibson TJ, Karplus K, Li W, et al. Fast, scalable generation of high-quality protein multiple sequence alignments using Clustal Omega. Mol Syst Biol. 2011 Jan;7(1):539.

8. Brown NP, Leroy C, Sander C. MView: a web-compatible database search or multiple alignment viewer. Bioinforma Oxf Engl. 1998;14(4):380–1.

9. The UniProt Consortium, Bateman A, Martin MJ, Orchard S, Magrane M, Agivetova R, et al. UniProt: the universal protein knowledgebase in 2021. Nucleic Acids Res. 2021 Jan 8;49(D1):D480–9.

10. Berardini TZ, Reiser L, Li D, Mezheritsky Y, Muller R, Strait E, et al. The arabidopsis information resource: Making and mining the “gold standard” annotated reference plant genome: Tair: Making and Mining the “Gold Standard” Plant Genome. genesis. 2015 Aug;53(8):474–85.

11. Amos B, Aurrecoechea C, Barba M, Barreto A, Basenko EY, Bażant W, et al. VEuPathDB: the eukaryotic pathogen, vector and host bioinformatics resource center. Nucleic Acids Res. 2022 Jan 7;50(D1):D898–911.

12. Trager W, Jensen JB. Human Malaria Parasites in Continuous Culture. Science. 1976 Aug 20;193(4254):673–5.

13. Ponnudurai T, Lensen AHW, Leeuwenberg ADEM, Meuwissen JHETh. Cultivation of fertile Plasmodium falciparum gametocytes in semi-automated systems. 1. Static cultures. Trans R Soc Trop Med Hyg. 1982 Jan;76(6):812–8.

14. Ponnudurai T, Lensen AHW, Van Gemert GJA, Bensink MPE, Bolmer M, Meuwissen JHETh. Infectivity of cultured *Plasmodium falciparum* gametocytes to mosquitoes. Parasitology. 1989 Apr;98(2):165–73.

15. Yang ASP, Waardenburg YM, Vegte-Bolmer M, Gemert GA, Graumans W, Wilt JHW, et al. Zonal human hepatocytes are differentially permissive to *Plasmodium falciparum* malaria parasites. EMBO J [Internet]. 2021 Mar 15 [cited 2023 Jan 27];40(6). Available from: https://onlinelibrary.wiley.com/doi/10.15252/embj.2020106583

16. Wittig I, Braun HP, Schägger H. Blue native PAGE. Nat Protoc. 2006 Jun;1(1):418–28.

17. Ran FA, Hsu PD, Wright J, Agarwala V, Scott DA, Zhang F. Genome engineering using the CRISPR-Cas9 system. Nat Protoc. 2013 Nov;8(11):2281–308.
