# Supplementary material for: Comparative Clustering (CompaCt) of eukaryote complexomes identifies novel interactions and sheds light on protein complex evolution
Source: PLoS Comput Biol. 2023 Aug 7;19(8):e1011090. doi: 10.1371/journal.pcbi.1011090 (PMC10434966; doi:10.1371/journal.pcbi.1011090)
Supplement: Attachment — Submitted filename: Response_to_reviewers.pdf [file pcbi.1011090.s004.pdf]

Response to reviewer's comments

We would like to thank the reviewers for their valuable comments. In the following section we address each of the reviewer's comments point by point, and describe the changes made to the manuscript. Line numbers correspond to those in the revised manuscript with highlighted changes.

Reviewer #1: The manuscript presents a new tool for deriving protein complex predictions from CF-MS experiments. I really like the approach of using orthology between species to try to build better interactomes. I have a few questions/concerns:

We thank reviewer 1 for their appraisal of the approach we developed and present in our manuscript .

1. What exactly is the input data? E.g., will CompaCt take output from PrInCe? I get that it takes pairs of proteins with some kind of likelihood score but more information is needed there.

We agree with the reviewer that the sections describing the exact nature of the input data needs further clarifications. We amended the relevant sections of the results, discussion and methods as follows.

results section, line 155-162: "CompaCt requires as input datasets with interaction scores between all protein pairs. Any numeric values representing interaction strength or likelihood that allows ranking each protein's interactors can be used (e.g., correlation, machine learning-based scores, etc.). In the specific application of CompaCt used in this project we have used Pearson correlation between the protein migration patterns resulting from complexome profiling as interaction score. To estimate whether two proteins have common interactors, we compare their sets of interactions that are ranked based on their interaction scores."

discussion, line 656-662:"However, recent work has suggested and demonstrated the effectiveness of several other metrics or machine learning-based scores to determine interactions (7,8,11,28,55). Here we refrained from using those, as our focus was on the method of integrating interaction datasets rather than identifying the best within-dataset interaction metric. Additionally, several of these metrics rely on external evidence and a reference set of known complexes, which are not available for some of the less well-studied species."

methods, line 680-684: "Any numeric metric or score that reflects interaction likelihood or strength and allows ranking of each protein's interactors can be used. Optionally, the raw element expression/abundance data (e.g.: protein abundances per fraction in case of complexome profiles) can be provided, in which case CompaCt automatically computes Pearson's correlation scores between element pairs."

2. The Foster group has shown that clustering interactome data to derive complex predictions is extremely susceptible to noise (https://pubmed.ncbi.nlm.nih.gov/33592499/).

How does CompaCt get around this issue? It appears that only a single type of clustering is applied, with no way to control for noise introducing errors

The reviewer raises an interesting point. We apologize for the oversight, and have added a paragraph to the discussion in which we address the susceptibility to noise inherent in protein-protein interaction networks, and discuss our approach to prioritizing robust and biologically relevant clusters in the discussion.
Discussion, line 591-598:"Stacey et al. have recently shown that clustering of protein-protein interaction networks is susceptible to noise (50). They found that part of the resulting clusters are stable and robust to noise while others are not, with the former more often biologically relevant, and propose a perturbation strategy to determine the stability of clusters. Rather than using a perturbation approach to identify likely biologically relevant and stable clusters, CompaCt leverages the fact that it combines multiple datasets to determine the consistency with which proteins and their orthologs from different datasets are clustered together, captured by the "cluster coherence" score."

3. How does CompaCt perform compared to other algorithms? I was expecting to see some side-by-side analysis demonstrating that this gives some advantage

To our knowledge CompaCt is unique in its ability to perform combined clustering of data from multiple complexomes in a manner that enables identification of taxon-specific complex subunits. Nevertheless, we agree with the reviewer that it would be valuable to compare its performance to methods that aim to identify protein complexes from protein interaction data representing a single complexome. As we have now clarified in our manuscript (see above) CompaCt can take any numeric pairwise protein interaction generated by other methods as input, including those generated by existing approaches, and performs integrative identification of complexes using these interaction scores. Therefore, we agree that it would be interesting to see how our method compares to the state of the art with regards to identifying protein complexes from protein interaction scores. To this end we compared the performance of our approach to clusteONE, a state of the art method that is commonly used to identify protein complexes from protein interaction data, when applied to the human complexome data used in this study. The results of this comparison are now described in the manuscript and visualized in a supplemental figure. A detailed description of the approach is described in the supplemental methods.

results paragraph, line 271-282: "To our knowledge CompaCt is unique in its ability to perform combined clustering of PPI data from multiple complexomes, while allowing the composition of clusters to vary per complexome, thus enabling identification of taxon-specific elements. However, to compare the performance of CompaCt with existing approaches that aim to identify complexes from a single complexome, we compare it to the performance of ClusterONE (27), a state-of-the-art method commonly used to identify protein complexes from protein interaction data (7–9,28). We applied ClusterONE to the human protein interaction datasets used as input for CompaCt. Supplemental Figure S1 shows the agreement of the ClusterOne output clusters with CORUM using optimized parameters, compared to the CompaCt results (Supplemental Figure S1, Supplemental Methods). CompaCt performance is comparable to ClusterONE when applied to the human complexome data, but outperforms ClusterONE when applied to the complete set of complexomes."

supplemental methods section: "To determine the performance of ClusterONE (v1.0) , it was applied to the eight H. sapiens complexome profiling datasets, using the parameter settings that yielded the highest performance. The optimal ClusterONE parameters were determined by computing the agreement of cluster performance for various parameters for a range of values for each parameter, while keeping the other parameters fixed (Supplemental Figure S1). The optimal parameter settings were as follows: overlap=0.96, density=0.8, haircut=1.0. As the penalty parameter did not have a significant effect on the performance it was kept at its default value of 2. In addition to the aforementioned ClusterONE algorithm parameters, different threshold values for inclusion of correlation score edges in the ClusterOne input were evaluated (Supplemental Figure S1). Retaining only edges with a correlation score of at least 0.96 resulted in the best performance."

Reviewer #2: The manuscript by Joeri van Strien, Felix Evers, Madhurya Lutikurti, Stijn L. Berendsen, Alejandro Garant, Geert-Jan van Gemert, Alfredo Cabrera-Orefice, Richard J. Rodenburg, Ulrich Brandt, Taco W.A. Kooij and Martijn A. Huynen describes a method named CompaCt that allows the comparison of complexome profiles across various experiments and different species. The authors describe very well the methodology of native protein preparation and separation before describing the methodological challenges to comparatively analyze multiple complexome profiles, that differ in preparation, separation and detection methods. By converting similarities between protein profiles within data sets in ranked lists of decreasing local similarity, CompaCt can compare profile similarities across various experiments. After filtering and normalization of the RBO score matrix, a network is generated and clustered by MCL. Metrics to interpret the relevance of specific proteins or clusters are provided as fraction_clustered and cluster coherence respectively. By including various data sets of different tissues/species, CompaCt can distinguish between consistently co-migrating proteins and spuriously co-migrating proteins, and provides interesting insights into new putative evolutionary conserved complexes.
The method presented by the authors is very interesting and intriguing and the manuscript is well written. However, there are some questions that remain unclear after reading the manuscript in its current form.

We would like to thank reviewer 2 for their positive appraisal of our manuscript and the method presented within.

Comments:

1. In "Comparing interactor profiles with rank biased overlap" it is stated that "the rbo metric is a set-based overlap metric, that assigns a degree of overlap between two ranked lists". Sets do not inherit any particular element order. It should be clarified, that the ranking is a key property for this rank similarity metric.

As suggested by the reviewer, we clarified the importance of the ranking of the lists rather than the content, in the relevant methods section, line 704-706: "RBO is a so-called set-based overlap metric, that assigns a value between 0 and 1 representing the degree of similarity between the ranking of two ranked lists of elements."

2. In Processing clusters: prioritizing clusters it is stated that "The total number of matches found within a supercluster is then divided by the number of possible matches given the cluster's composition, to get the fraction of possible matches." It is ambiguous how the total number of matches is determined. Is it the number of possible edges, or is the orthologue distribution taken into account? How is a coherence of 0.65 obtained for rubisco given the supplemental data. While the point size and x-axis location match the table, the coherence score is not clear.

As suggested by the reviewer, we revised the description of the fraction of possible matches to remove ambiguity, and provided the rubisco complex cluster as an example to illustrate and clarify how the possible number of matches is computed, providing tables with the composition and match counts of this supercluster cluster.

line 784-798: "The total number of matches found within a supercluster is then divided by the total number of possible matches given the cluster's composition, to get the fraction of possible matches**.** Consider that a cluster contains n proteins from dataset A, and k proteins from dataset B. The number of possible matches would then be the equal to the minimum value of n and k. In the case where a cluster contains proteins from more than two datasets, the possible number of matches is the sum of possible matches between each dataset pair. In addition to computing a coherence score for each complete supercluster, CompaCt computes the fraction of possible matches for each specific subcluster, to reflect the coherence of each cluster in that specific system. As an example, the computation of the fraction of possible matches for the CompaCt supercluster containing the rubisco complex is illustrated in Table 3. The composition of this supercomplex is shown in Supplementary Table S2. To calculate the fraction of possible matches the total number of actual matches (46) is divided by the total number possible matches (71), resulting in a score of 0.648. To remove clusters that are not likely to be biologically relevant, CompaCt performs a filtering step, retaining only clusters with a minimum of 2 matches as well as having at least one protein with a fraction clustered of at least 0.5."

3. Please elaborate, why both Arabidopsis samples in the first 3 inclusion steps are the most detrimental for the MMR, but afterwards perform as top candidates in recovering CORUM complexes.

To address the point the reviewer raises, we added a paragraph to the results that hypothesizes how the beneficial effect of including additional datasets varies depending on the included complexome as well as on the stage in which the datasets are included in the analysis (line 260-269): "Notably, while generally the recovery of human complexes improves as additional data are included in the analysis, the effect varies per complexome and across inclusion stages. Given the heterogeneity of the analysed data (e.g., resolution, number of replicates, number of detected proteins, sample processing, evolutionary distance from human etc.), we are unable to determine from this which dataset features contribute most to improved recovery of complexes. With regards to varying benefits depending on the stage at which data is included, it is possible that lower-resolution datasets (such as those from A. thaliana) might initially be detrimental to the formation of well-defined modules in the hypernetwork, while they might add some value in the presence of sufficient high-resolution data to form clearly defined modules."

Minor:

o In SF2_selected_clusters.xlsx the sheet “45_selected_clusters”, Column “fraction_present” Float decimal points are corrupted

We thank the reviewer for pointing this out, and now provide an updated supplemental file where this issue is resolved.
